# A cable driven robotic palpation system with contact force sensing based on cable tension observation

**DOI:** 10.1002/rcs.2435

**Published:** 2022-07-03

**Authors:** Chikweto Francis, Taiga Sato, Takeshi Okuyama, Mami Tanaka

**Affiliations:** ^1^ Department of Biomedical Engineering Graduate School of Biomedical Engineering Tohoku University Sendai Japan; ^2^ Department of Robotics Graduate School of Engineering Tohoku University Sendai Japan

**Keywords:** contact force, prostate cancer, robotic palpation, tissue stiffness

## Abstract

**Background:**

Prostate Cancer screening based on manual palpation is subjective. Robotic palpation systems can objectively acquire stiffness conditions of the prostate.

**Methods:**

A 2DoF cable driven robotic system for prostate palpation is proposed. An indirect method to estimate the contact force based on cable tension observation is presented. Kinematic models and a joint angle estimation method to determine the tip position of the probe are derived. Positioning accuracy was verified using an optical marker tracking system and by displacement measurement, respectively. The contact force estimation method was validated on silicone phantom samples.

**Results:**

A good consistence between the estimated and measured contact force was observed. The contact force was correlated with the elastic modulus of each silicone phantom. There was also a good agreement between the theoretical and the measured tip position.

**Conclusion:**

In the proposed palpation system, the indirect contact force estimation method is viable and holds potential for the stiffness assessment of the prostate. The tip position vital for palpation can be determined through estimated joint angles.

## 1 INTRODUCTION

Prostate cancer is the second frequently diagnosed cancer in men and the fourth most commonly occurring cancer overall.^1^ Early detection and staging of prostate cancer are non‐trivial. Standard clinical screening methods for prostate cancer include a digital rectal examination (DRE) and prostate specific antigen (PSA) blood tests.^2^, ^3^ Following elevated PSA levels and positive DRE results, image guided biopsy which is serves as a gold standard for prostate cancer diagnosis is performed. Prostate cancer mainly arises in the posterior region. Therefore, it can be accessible and detectable from this region through palpation. Tumours are often stiffer and irregular than normal tissue. During DRE, a clinical examiner inserts a gloved and lubricated finger into the patient's rectum and induces palpation motions on the posterior surface of prostate gland. The presence or absence of tumours can be inferred from the tactile cues on the fingertip in response to stiffness differences between normal and cancerous tissues.^4^ DRE is readily available, cost effective and possess lower risk. If combined with PSA blood tests, it could enhance the early detection rate of prostate cancer.^5^ However, DRE results are subjective, lacking objective and quantitative information. The sensitivity is highly dependent on the examiner's skill level and experience.^6^, ^7^ Evidence from several studies suggests that DRE may not significantly reduce mortality, instead may result in a high number of false positives, leading to unnecessary invasive diagnostic tests which can lead to erectile dysfunction and overdiagnosis.^8^, ^9^, ^10^

In order to obtain mechanical properties of the prostate tissue, several modalities have been developed in a number of studies.

These methods encompass direct mechanical properties characterisation based on indentation, compression, and tensile tests. Indentation tests performed by applying a force on a soft tissue and then recording the resulting deformation are a common approach to determine tissue elasticity.^11^ However, many such systems proposed in most studies are bulky and limited to ex vivo setup conditions. This is mainly due to the size constraints of the force sensors used. The need to develop cost effective robotic palpation systems which are potentially ideal for in vivo deployment is nontrivial. Robotic palpation systems can provide objective and quantitative information for the better understanding of the underlying morphological changes in the tissues using mechanical properties as potential biomarkers for tumours. In addition to the methods mentioned above, elastography and indirect modalities based on optical fibres have also been explored in the assessment of mechanical characteristics of the prostate tissue.^12^, ^13^, ^14^, ^15^, ^16^

This study is aimed at developing a cost effective robotic palpation system for the assessment of mechanical characteristics of the prostate. Contact force and position information is of great importance in robotic palpation. In many robotic systems, force sensors embedded at the tool end and rotary encoders in the joints are used for the measurement of the contact force and the joint angles, respectively. However, due to the size constraints of the proposed probe, this paper proposes the implementation of an indirect contact force estimation method based on cable tension measurement. Furthermore, it highlights a method to estimate the joint angles, which is essential for position estimation of the tip.

The paper is organised in the following manner: Subsection 2.1 in materials and methods provides the design details of the palpation system. Subsection 2.2 gives the details about the kinematic models relating the joint angles to the tip position of the probe, a model to describe the mapping of the joint angles to motor angular displacements, and joint angle estimation using the measured spring displacement. Subsection 2.3 introduces a method to estimate the contact force. Section 3 details experiment setups and results for both position measurement and contact force estimation. A discussion and directions for future work are presented in Section 4.

## 2 MATERIALS AND METHODS

### 2.1 Robotic palpation system design

**FIGURE 1:**
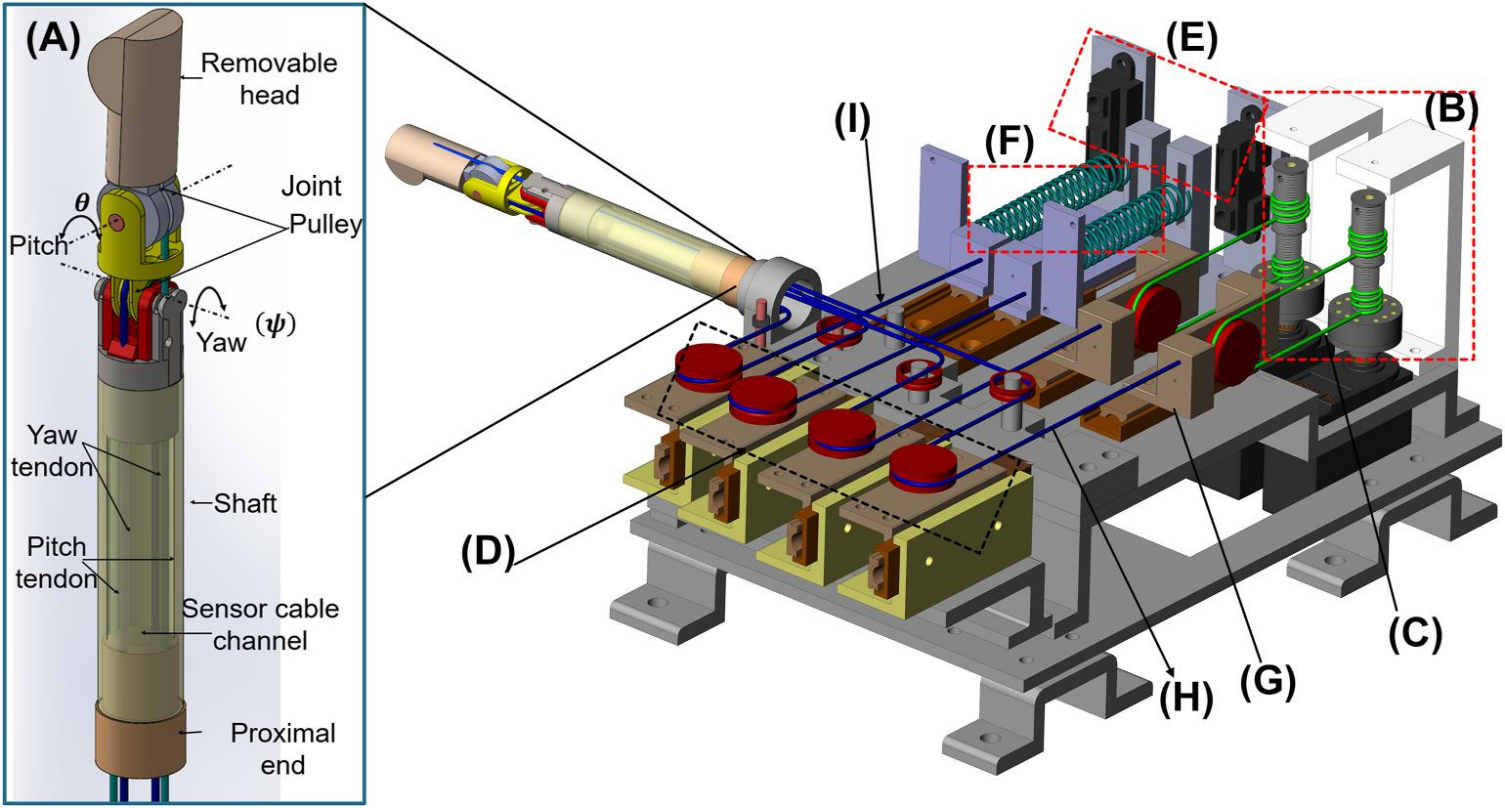
A computer aided drawing model of the robotic palpation system: (A) palpation probe, (B) differential pulleys mounted on the motor shafts, (C) primary cable, (D) cable tension measurement subunit, (E) displacement measurement subunit, (F) return springs, (G) sliding mechanism, (H) driving wire cable (tendon), (I) returning wire cable (tendon)

Figure 1 shows a computer aided drawing model of the proposed robotic palpation system; It is comprised of a Two Degrees of Freedom (2DoF) cable driven palpation probe, a sensing unit, and a driving mechanism unit consisting of stepping motors (yaw and pitch motors), differential pulleys, return springs and sliding mechanisms. For in vivo palpation of the prostate, the probe specifications must allow entry through a constrained entry point (anus). Once aligned with the target, the 2DoFs of the probe can be used to initiate palpation and should cover at least 40 × 30 mm area, the average size of the prostate.^17^ There is also need for an extra robotic manipulator to shuffle the probe at least 100 mm from the entry point and to position the probe tip at an arbitrary point along the path of the rectum for more dexterity.^18^ This study does not cover the manipulator. A probe is to be covered with a lubricated glove and shuffled into the rectum of a patient. By controlled palpation, the tip of the probe applies pressure on the prostate. The contact force and pressing depth can be obtained and used for stiffness characterisation of the prostate. The contact force between the tip and the palpated sample is estimated indirectly by measuring the difference in the tension between the driving and the returning cable of each joint, using load cells positioned away from the probe.

#### 2.1.1 Palpation probe

The palpation probe shown in Figure 1A is essentially comprised of a 2DoF articulating wrist consisting of the yaw and pitch revolute joints with their axes of rotation perpendicular to each other, a hollow shaft, and a removable head cover as a tool end. Two steel (SUS304, *ϕ* = 0.45 mm) wire cables (driving and returning wire cables) actuate each joint independently. The cables are routed along the probe's plane of symmetry via four cable guide holes (*ϕ* = 1.2 mm) located 10 mm radially away from the centre of the wrist and wrap around each joint pulley. For easy controllability, the joints are mechanically decoupled as much as possible. The ends of the driving wire cable are fixed to a sliding mechanism which is connected to differential pulleys via a primary wire and to a joint pulley, respectively. The returning wire cable is fixed between the joint pulley and the return spring. The wrist also features non movable guide arcs on the yaw part of the wrist to prevent acute bending of the cables when the probe yaws.^19^ This ensures durability of the cables, simpler construction of the probe with few moving parts. The probe's dimensions were set to 14 mm outer diameter and 135 mm length.

#### 2.1.2 Driving mechanism unit

The driving mechanism unit embodies two cable–pulley transmission systems to drive each joint independently. Each system consists of two 3D printed concentric differential pulleys of radii 6 and 7 mm mounted on a motor shaft (P‐PMSA‐U42D2) as shown in Figure 1B, a sliding mechanism and a pretensioned return spring (*k* = 0.15 N/mm, 55 mm free length, 48.5 mm tensile limit). The sliding mechanism is connected to the differential pulleys via a primary wire cable, and to the joint pulley by a driving cable. A return spring serves as a passive actuator. When the differential pulleys move clockwise and counterclockwise, each primary wire winds and unwinds from them, respectively. This in turn translates the sliding mechanisms back and forth, respectively, leading to the actuation of the joints. The winding amount of the driving and returning cables to the joint pulley depends on the angular displacement of each motor.

#### 2.1.3 Sensing unit

The sensing unit is comprised of a cable tension subunit and a displacement measurement subunit shown in Figure 1D,E. The cable tension measurement subunit consists of four one‐axis load cells coupled to cable routing pulleys. The tension in each cable is measured by each respective load cell. The home position cable tension is set to 5.5 N. The displacement of each return spring is measured by a distance sensor (Sharp GP2Y0A41SK0F) of the displacement measurement subunit. The size of the probe limits the integration of a rotary encoder in each joint for angle measurement. Therefore, the measured displacement is essentially used for estimating the joint angles of the probe. In a practical setup, the tip position can then be determined from the estimated joint angles.

### 2.2 Robot kinematics

#### 2.2.1 Forward kinematics

**FIGURE 2:**
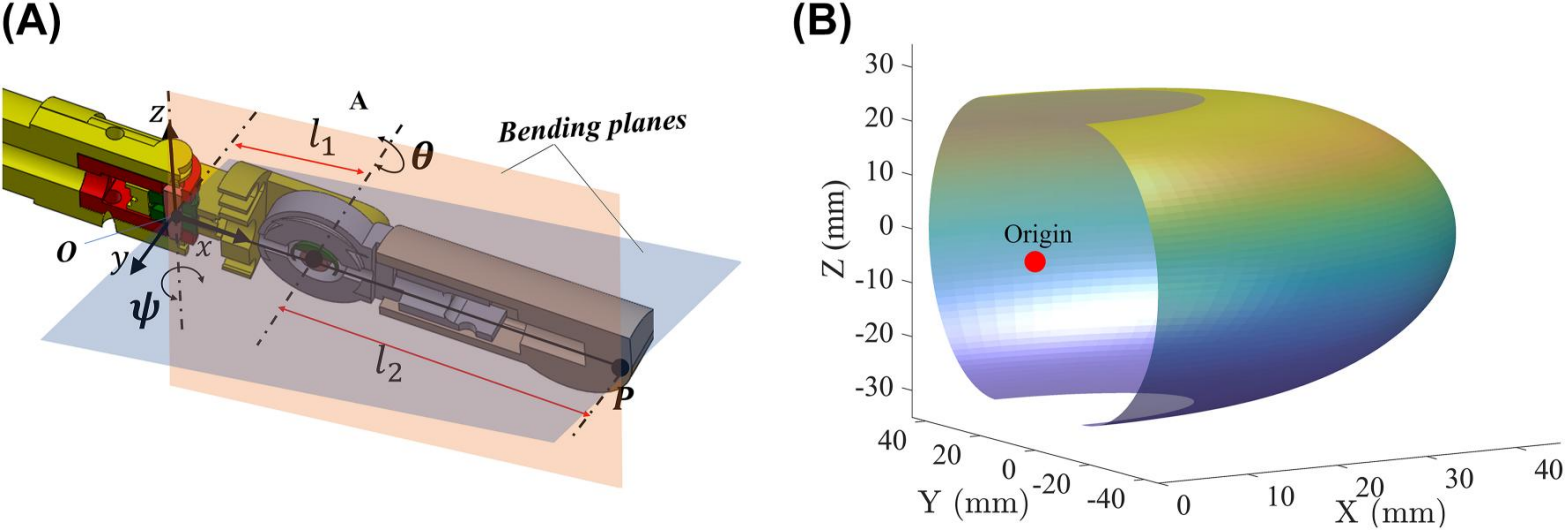
Kinematic representation: (A) probe's kinematic parameter description and (B) workspace for the tip *P* of the probe

The articulating wrist of the probe illustrated in Figure 2A is modelled using standard robot kinematics, where *l*_1_ = 15 mm and *l*_2_ = 28 mm correspond to the offset links between the yaw and pitch joint axes, and between the pitch joint and the probe's tip, respectively. The range of motion for the joint angles  *ψ* and *θ* is [−90°,+90°]. A position vector *P* with respect to the origin *O* can be expressed by the Equation (1). By using this equation, the coordinates for the tip of the probe can be defined as a function *P*(*θ*, *ψ*).The resulting workspace of the probe's tip is presented in Figure 2B.

(1) $$P\left(\theta ,\psi \right)=[\begin{array}{l} x\\ \begin{array}{l} y\\ z \end{array} \end{array}]=\left[\begin{array}{l} \left(l_{2}\cos\theta +l_{1}\right)\cos\psi \\ \begin{array}{l} \left(l_{2}\cos\theta +l_{1}\right)\sin\psi \\ l_{2}\sin\theta \end{array} \end{array}\right]$$

#### 2.2.2 Joint actuation

**FIGURE 3:**
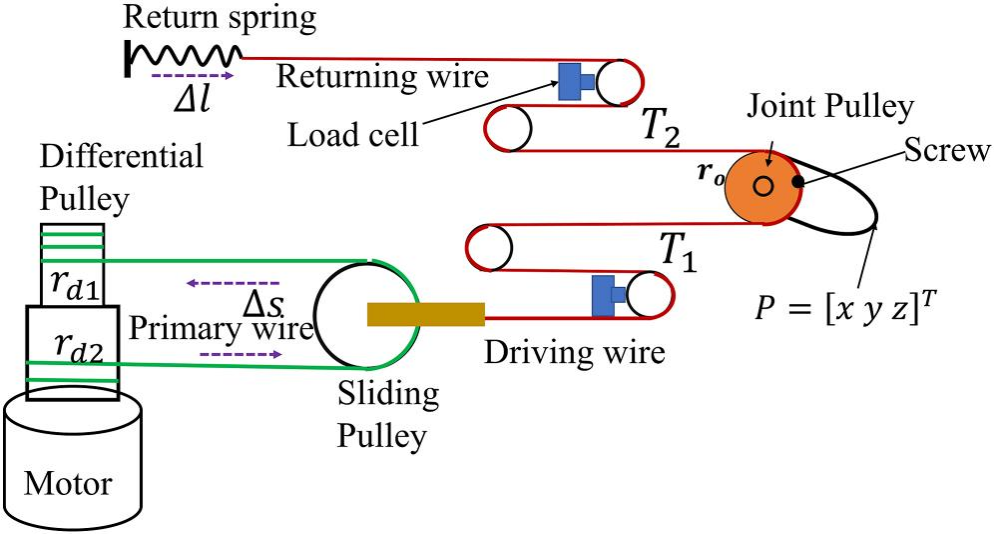
Wire cable tension measurement configuration and joint actuation principle

Each joint is actuated by a driving cable which is actively driven by concentric differential pulleys mounted on a motor shaft and coupled to a sliding mechanism via a primary wire cable, and by a returning wire cable, passively driven by a return spring as shown in Figure 3. The change in length **Δ*s*** of the primary wire cables can be calculated from the yaw and pitch motor angular displacement $\omega ={\left[\omega_{y}\;\omega_{p}\;\right]}^{T}$ as,

(2) $$\Delta s=\left(\frac{r_{d2}-r_{d1}}{2}\right)\left[\begin{array}{l} \omega_{y}\\ \omega_{p} \end{array}\right],$$

where *r*_*d*1_ and *r*_*d*2_ are the differential pulleys radii. This change in length **Δ*s*** results in the rotation of the joint pulleys by ***q*** = [*θ* *ψ* ]^*T*^. The yaw and pitch motor rotations are essentially calculated from the two joint variables ***q*** using the following expression.

(3) $$\omega \;=\left(\frac{2r_{o}}{r_{d2}-r_{d1}}\right)q=Dq=D\left[\begin{array}{l} \psi \\ \theta \; \end{array}\right],$$

where *r*_*o*_ is the radius of each joint pulley and is *D* is the reduction ratio. From Equation (3), a very high reduction ratio can be obtained by varying *r*_*d*1_ and *r*_*d*2_ during the design process.

#### 2.2.3 Joint angle estimation

The measured displacement **Δ*l *** of the return springs is equivalent to the change in length **Δ*s*** of the primary wire cables. Hence, **Δ*l*** is related to the change in the pose of the probe which is the function of the joint angles *ψ* and *θ*. By using **Δ*l***, the joint variables $\bar{q\;}$ can be approximated as

(4) $$\bar{q}={\left[\;\bar{\psi \;}\;\bar{\theta }\;\right]}^{T}=\frac{\Delta l}{r_{o}},$$

where $\bar{\psi }$ and $\bar{\theta }$ are the estimated yaw and pitch joint angles. Consequently, the actual position of the probe's tip can be approximated by using Equations (4) and (1) in a practical setup.

### 2.3 Contact force estimation

It is desirable that all sensors are placed far away from probe's tip.

In this way, the probe's head cover can easily be disposed or exchanged. Furthermore, it minimises the chances of contamination of sensors by biological fluids, offers easier sterilisability as well as minimal costs of replacing sensors. Due to the size constraints of the palpation probe, installation of most commercially available force sensors at the at the tip of the probe may not be feasible. Therefore, in this system, the four wire cables used for changing the pose of the probe are also simultaneously utilised for indirectly estimating the contact force at the tip of the probe.

The two joints have a similar wire cable tension measurement and joint actuation principle. The antagonistic configuration of the driving and the returning wire cable actuates each joint pulley as shown Figure 3. The cable tensions  *T*_1_ and  *T*_2_ in the driving and the returning cables of each joint are measured by single axis load cells. Contact force is then determined indirectly through the measured cable tensions and pose information. The tip position *P* of the probe and the joint variables (*ψ*,*θ*) are associated with each other through a Jacobian which relates joint velocities to end effector velocities of the probe as follows,

(5) $$\dot{P}=\left[\begin{array}{l} \dot{x}\\ \dot{y}\\ \dot{z} \end{array}\right]=J\left[\begin{array}{l} \dot{\psi }\\ \dot{\theta }\; \end{array}\right],$$

where the Jacobian *J* for this robotic palpation probe is given by

(6) $$J=\left[\begin{array}{ll} \frac{\partial x}{\partial \psi } & \frac{\partial x}{\partial \theta }\\ \frac{\partial y}{\partial \psi } & \frac{\partial y}{\partial \theta }\\ \frac{\partial z}{\partial \psi } & \frac{\partial z}{\partial \theta } \end{array}\right]=\left[\begin{array}{cc} -\sin\psi \;\left(l_{1}+l_{2}\;\cos\;\theta \right) & -l_{2}\;\sin\;\theta \;\cos\psi \\ \cos\psi \;\left(l_{1}+l_{2}\;\cos\;\theta \right) & \;-l_{2}\;\sin\;\theta \;\sin\psi \\ 0 & l_{2}\;\cos\;\theta \end{array}\right]$$

Using the principle of virtual work, a method for estimating the contact force at the tip of the probe through tension measurement of the driving wire cables is presented. The difference in the measured tension in the driving and returning cables of each joint yields a joint torque *τ* = (*T*_1_ − *T*_2_) × *r*_*o*_.

In general, joint torques are related to contact forces or the wrench through a Jacobian. However, the Jacobian above is non‐square and there are only two joint torque variables associated with this probe. Therefore, using singular valued decomposition, a Moore‐Penrose pseudo inverse $J^{+}={\left(J^{T}J\right)}^{-}J^{T}$ of the Jacobian matrix is instead found.^20^ The relationship between the contact force *F = *[*F*_*x*_ *F*_*y*_ *F*_*z*_]^*T*^ at the tip of the probe and the joint torques estimated through cable tension measurement can be expressed as

(7) $$F=-{\left(J^{T}\right)}^{+}\tau$$

The accuracy of this indirect contact force sensing method can be validated by comparing the estimated results with the ground truth contact force, measured for example, by using a three axis loadcell.

## 3 EXPERIMENTS

In order to verify the positioning accuracy as well as to validate both the joint angle estimation and the indirect contact force sensing methods of the proposed robotic palpation system, various experiments were conducted.

### 3.1 Probe's tip position measurement and joint angle estimation

**FIGURE 4:**
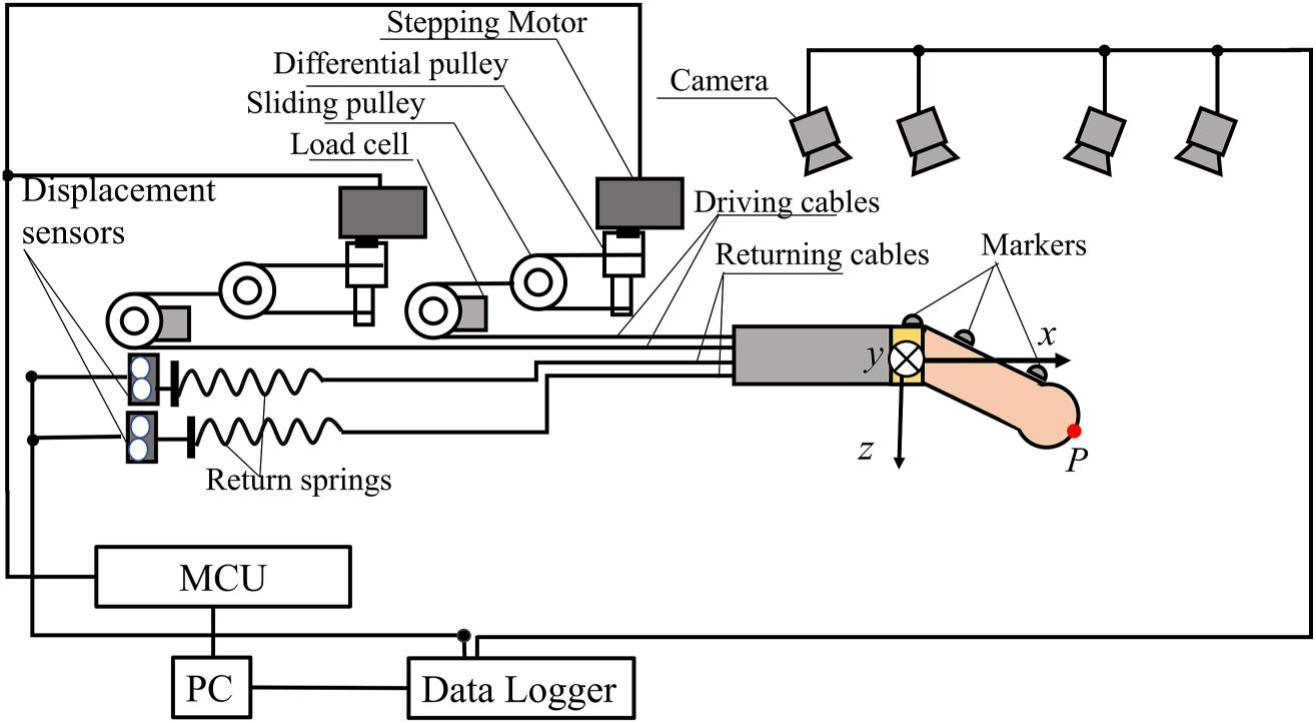
Experimental setup for position measurement using an optical marker tracking system

In this section, the tip positioning accuracy and the joint angle estimation method for the proposed palpation probe were validated by two different approaches: by optical marker tracking and by measuring the displacement of the return spring on one of the passive sides of the driving mechanism unit. Figure 4 shows an experimental setup for measuring the tip position *P* of the probe using an optical marker tracking system. Position measurement was achieved by tracking infrared reflective markers installed on the probe using a 3D motion capture system (VICON) with four infrared cameras (MX‐T160).The sampling rate was set at 100 Hz. An Arduino Mega microcontroller board was used for motor control. Experimental conditions were set for the yaw *ψ* and pitch *θ* joint variables according to the predefined five (5) trajectories. The yaw *ψ* angle was varied between −40° and 40° in increments of 20°. For each *ψ* condition, the pitch *θ* angle was varied from 0° to 80° in 10° increments. The obtained probe's tip position results were then compared with the theoretical values calculated using Equation (1), in order to determine the positioning error for each trajectory.

Tip position measurement using the optical marker tracking system may not be feasible in a practical clinical setup. In this case, the position can alternatively be obtained by estimating the joint angles using the measured displacement of the return springs. Therefore, a second position measurement experiment was setup, in which the displacement of the return spring was measured using a distance sensor (Sharp GP2Y0A41SK0F) in order to estimate the joint angle. Since the two joints are mechanically decoupled, only the pitch joint angle was investigated. The yaw angle *ψ* was set to 0° and the pitch *θ* was varied between 0° and 80° in 20° increments. An exponential moving average filter was applied to the sensor output for noise filtration. Using Equation (4), the pitch angles were estimated and compared with the reference input angles commanded by the user. Consequently, by using the estimated and the reference input pitch angle values in Equation (1), the estimated and theoretical z‐position values of the probe's tip were calculated, respectively.

#### 3.1.1 Position measurement results

**FIGURE 5:**
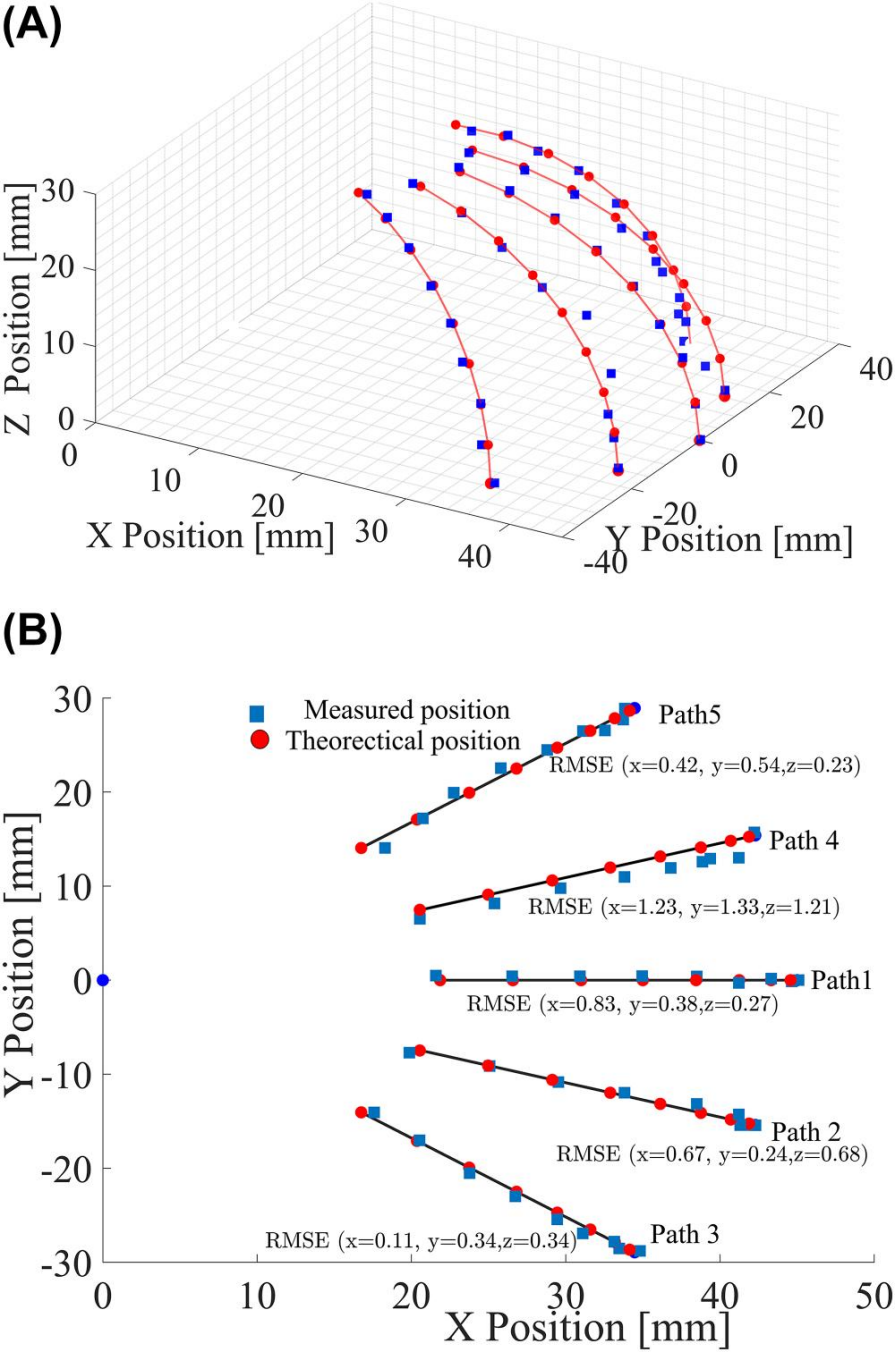
Theoretical and measured *P* position. (A) 3D view and (B) Top view with root mean squared error (RMSE) values for each trajectory

Figure 5 shows a comparison between theoretical and measured tip positioning results in the workspace subregion for which five (5) trajectories were considered. The results show that there was a relatively good agreement between the measured and theoretical values for all the 5 trajectories ψ∈[−40°,−20°,0,20°,40°] as the probe pitched between 0° and 80°. As shown in Figure 5B, the smallest root mean squared error (RMSE) values in the three directions were *x* = 0.11 mm, *y* = 0.24 mm and *z* = 0.23 mm. However, path (4) had relatively the largest RMSE values of (*x* = 1.23 mm, *y* = 1.33 mm, *z* = 1.21 mm).

The probe is expected to perform palpation by applying a pressing force on the tissue sample mainly in the *z‐*axis direction. Therefore, the positioning accuracy and contact force in this direction are particularly important. The RMSE margin reported shows that the positioning accuracy for this system may be sufficient enough for prostate palpation for this range of motion, given that for all the five palpation trajectories investigated, the largest RMSE was less than 1.40 mm.

**FIGURE 6:**
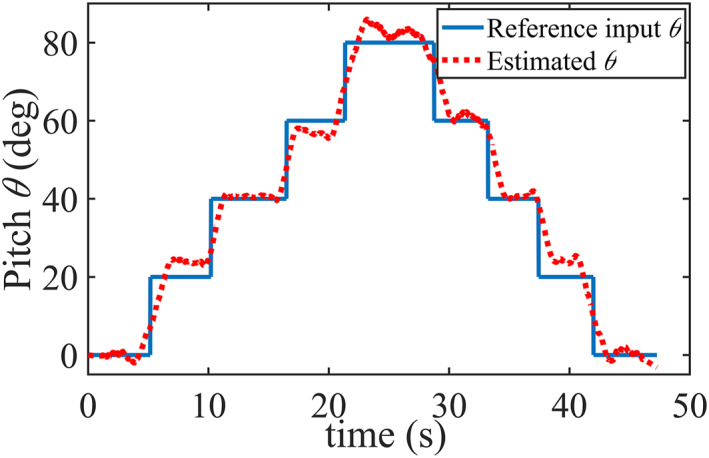
Comparison between the reference input pitch and estimated pitch angles derived from displacement measurement

A comparison of angular results between the reference input angles commanded by the user and the estimated *θ* angles calculated from displacement sensor output are given in Figure 6. The RMSE value was found to be 4.50°. The system is, therefore, capable of estimating and tracking the joint angles without the usage of a rotary encoder in the joints.

**FIGURE 7:**
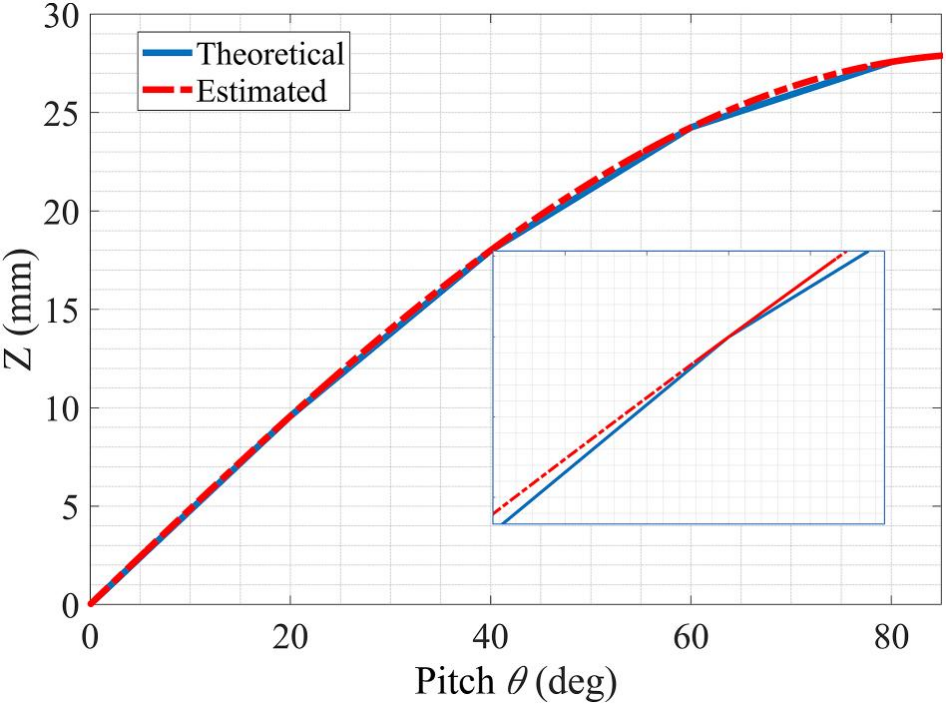
Comparison between the theoretical fingertip *z* coordinate and the estimated values derived from the estimated pitch angle

By using the estimated and reference input *θ* angles in Equation (1), both the theoretical and estimated tip z‐position values were determined, respectively. The results are given in Figure 7. The estimated and theoretical *z‐*position values show a good agreement the with mean absolute error of 1.05 mm. However, for very small displacements the distance sensors in this study may not be suitable for use. They can be replaced by other position sensors with a much higher resolution to improve the tip position estimation capability.

### 3.2 Contact force estimation

Prior to contact force estimation experiments, three silicone phantom samples (KE‐1300T,Shin‐Etsu,JAPAN) with different stiffness states (soft, medium, hard) were made in order to simulate three prostate tissue stiffness conditions; benign prostatic hyperplasia, normal, and cancer.

**FIGURE 8:**
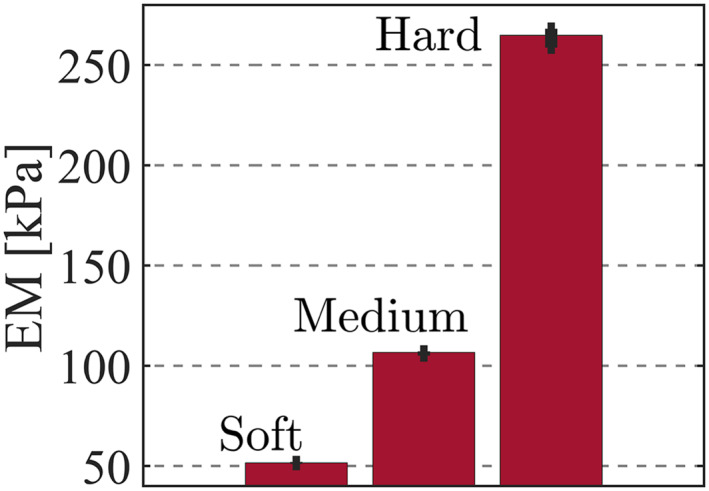
Elastic modulus (EM) of each silicone sample determined by indentation tests

The size of each sample was (40  × 20 × 20 mm). The elastic modulus (EM) of each sample was obtained by performing several indentation tests using YAWASA MSES‐0512. The mean EM of each sample was confirmed to be 52 kPa, 106 kPa and 264 Kpa,respectively, as shown in Figure 8. The EM values of the samples were larger than the reported EM for the prostate tissue in indentation test based studies such as those reported by Carson et al; 36.8 kPa, 41.1 kPa and 135.0Kpa for benign prostatic hyperplasia (BHP), normal and cancer conditions, respectively.^21^ However, this did not impede the purpose of the study.

The proposed probe has a unique joint mechanism. Pivoting about the yaw axis also results into bending of the pitch cables which can affect the initial cable tension in the two cables. A considerable change in tension in cables due to this yaw motion can adversely affect the force estimation accuracy. Circular guide arcs were therefore, optimally positioned on the yaw portion of the probe to minimise the acute bending. Prior to contact force estimation experiments on silicone samples, an experiment was carried out to investigate the effect of the yaw *ψ* motion on the tension of the pitch cables by varying the *ψ* between −80° and 80° in 20° increments whilst keeping *θ* at 0°. For each yaw condition, the tensions T1 in the driving cable and T2 in the returning cable were measured for 6 seconds, and the mean value was found. The initial cable tension at home position (*θ* = 0°, *ψ* = 0°) was set to 5.5 N. Ideally the yaw motion must exhibit little to no influence on the pitch cable tension; the tension must remain unchanged or slightly drift from the tension at home position.

**FIGURE 9:**
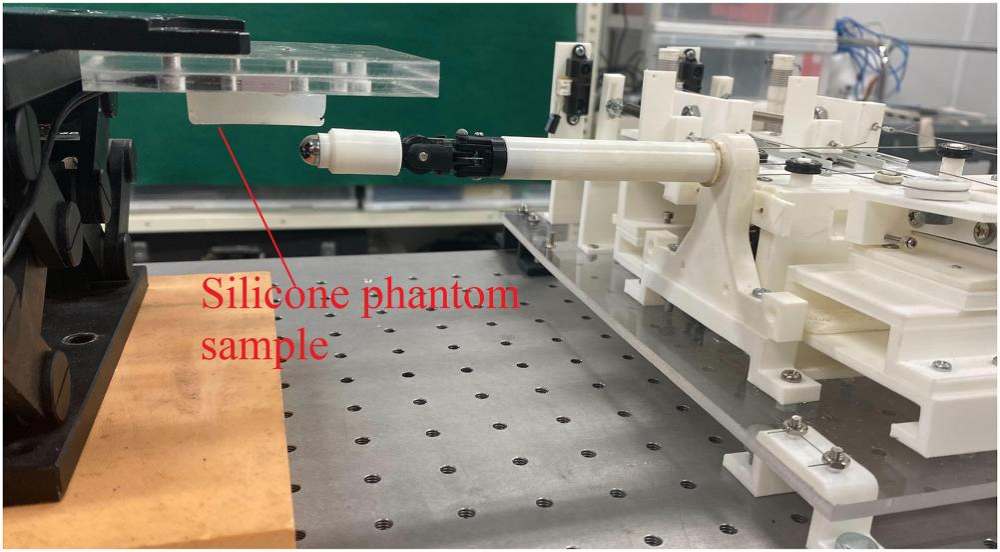
Physical experimental setup. A silicone phantom sample mounted on a *z*‐axis stage during palpation experiments

**FIGURE 10:**
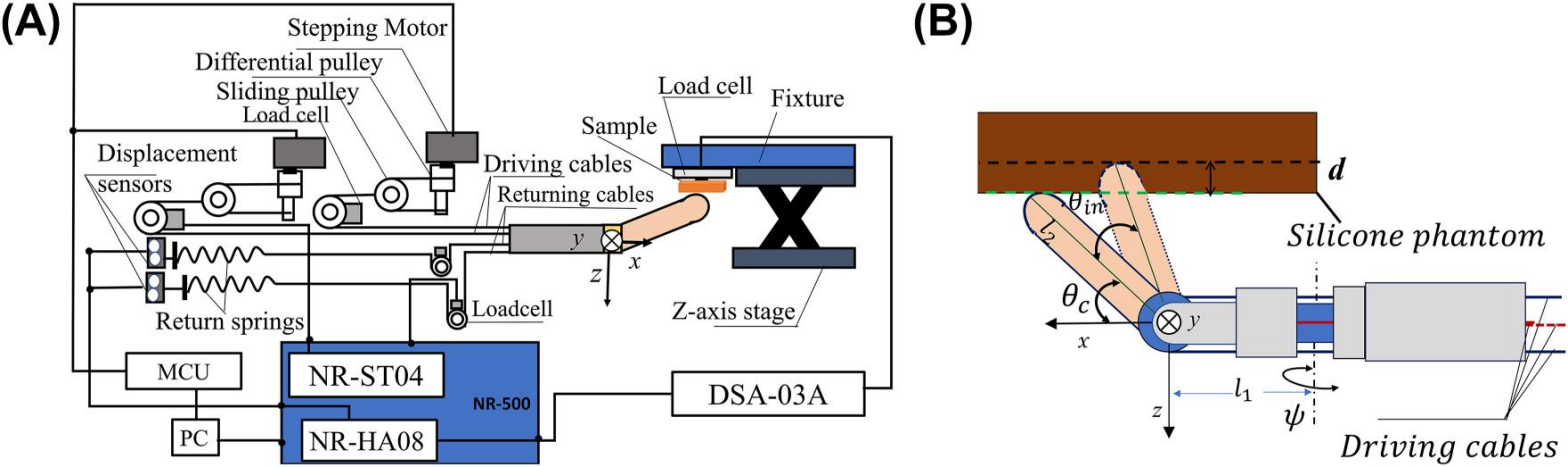
(A) Experimental setup for contact force estimation; (B) contact and indentation angle conditions

Figure 9 shows the actual experimental setup for indirect contact force sensing in which each silicone sample was palpated. On the other hand, Figure 10A shows a schematic representation of the entire experimental setup. A sample was fixed on an acrylic plate, and a three axis load cell (Tec Gihan, USL06‐H5‐50N‐C) was mounted above it in order to measure the ground truth contact force. The silicone sample, acrylic plate, and the three axis load cell were then fixed to a *z*‐axis stage. The loadcell was connected to a strain amplifier (DSA‐03A) and to analog channels (NR‐HA08) of the data acquisition device (NR‐500‐ KEYENCE).The tension in each wire cable was measured using a single‐axis load cell (KYOWA LMB‐A‐50N) connected to a strain amplifier (NR‐ST04) of the same data acquisition device mentioned above. To change the initial contact angle, the vertical position of the silicone sample was varied by adjusting the stage.

Experimental conditions were set based on the pitch *θ* and yaw *ψ* angles. Palpation was performed by changing the pose of the probe to the desired states. The initial contact pitch angle *θ*_*c*_ between the sample and the tip of the probe was set at 10° and 20° consecutively. *ψ* was set at 0° and 20°. For each sample, the pose of the probe was changed to satisfy each *ψ*, *θ*_*c*_, as well as the indentation angle *θ*_*in*_ palpation condition as shown in Figure 10B. After setting the initial contact angle, the position of the sample was adjusted on the *z*‐axis stage, such that the tip of the probe and the sample were in contact with each other. The probe was then pushed into the sample by *θ*_*in*_ = 10°. The tension in each wire cable and the 3‐axis load cell output were measured for 5 s after the probe stopped, and the average values were found. The procedure was repeated for *θ*_*in*_ = 20° condition. From this, the joint torque ***τ*** during contact was calculated and then together with the bending state (*ψ*, *θ*) information, were used in Equation (7) to indirectly estimate the contact force. In order to minimise measurement errors as much as possible, the measured tension by each load cell before initial contact with the sample was adjusted to 0N as a threshold value.

Depending on the pose of the probe and the pseudo inverse of the Jacobian the contact force **F** = [*F*_*x*_ *F*_*y*_ *F*_*z*_]^T^ can be estimated, provided the two joint torques are observed through the measured tension in all the four cables. In this study however, only the cable tension in the driving and returning cables associated with the torque on the pitch joint was investigated, since the dominant pressing force during palpation was due to the pitch motion.

#### 3.2.1 Contact force results

**FIGURE 11:**
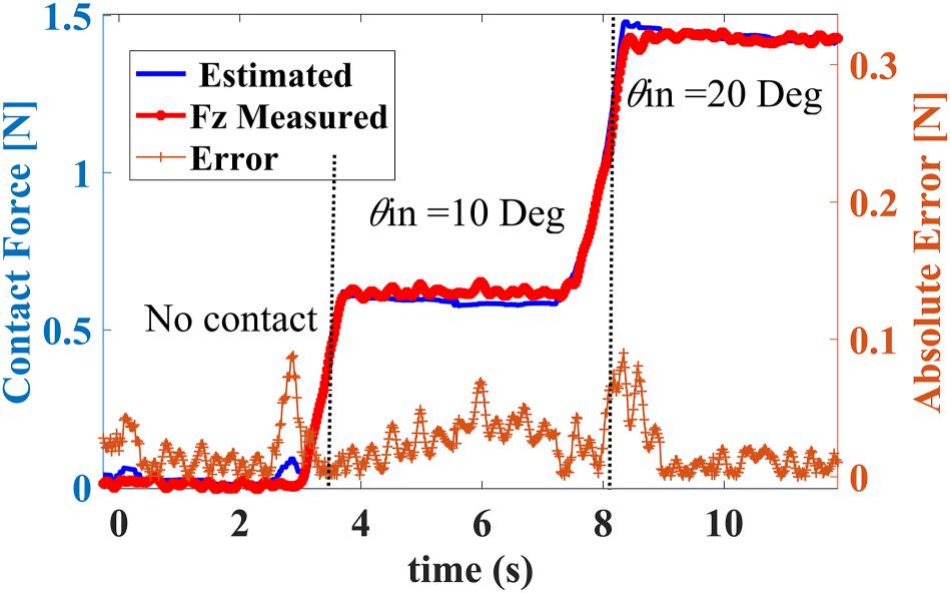
Results for the estimated and ground truth contact force for the hard silicone phantom under (*ψ* = 20°,*θ*_c_ = 20°) palpation conditions

For every palpation experiment on the samples, temporal domain measured cable tension and ground truth contact force data were acquired by the respective load cells. By using cable tension and Jacobian information, the contact force was indirectly estimated. Thereafter, the absolute error between the estimated and measured contact force was determined. Figure 11 shows the results for the measured and estimated contact force on a hard silicone phantom for *ψ* = 20° condition. Relatively good estimation results for contact force were achieved with 0.098 N maximum absolute error. The contact force increased from 0.62 to 1.82 N as the indentation angle *θ*_*in*_ was increased from 10° to 20°, respectively.

**FIGURE 12:**
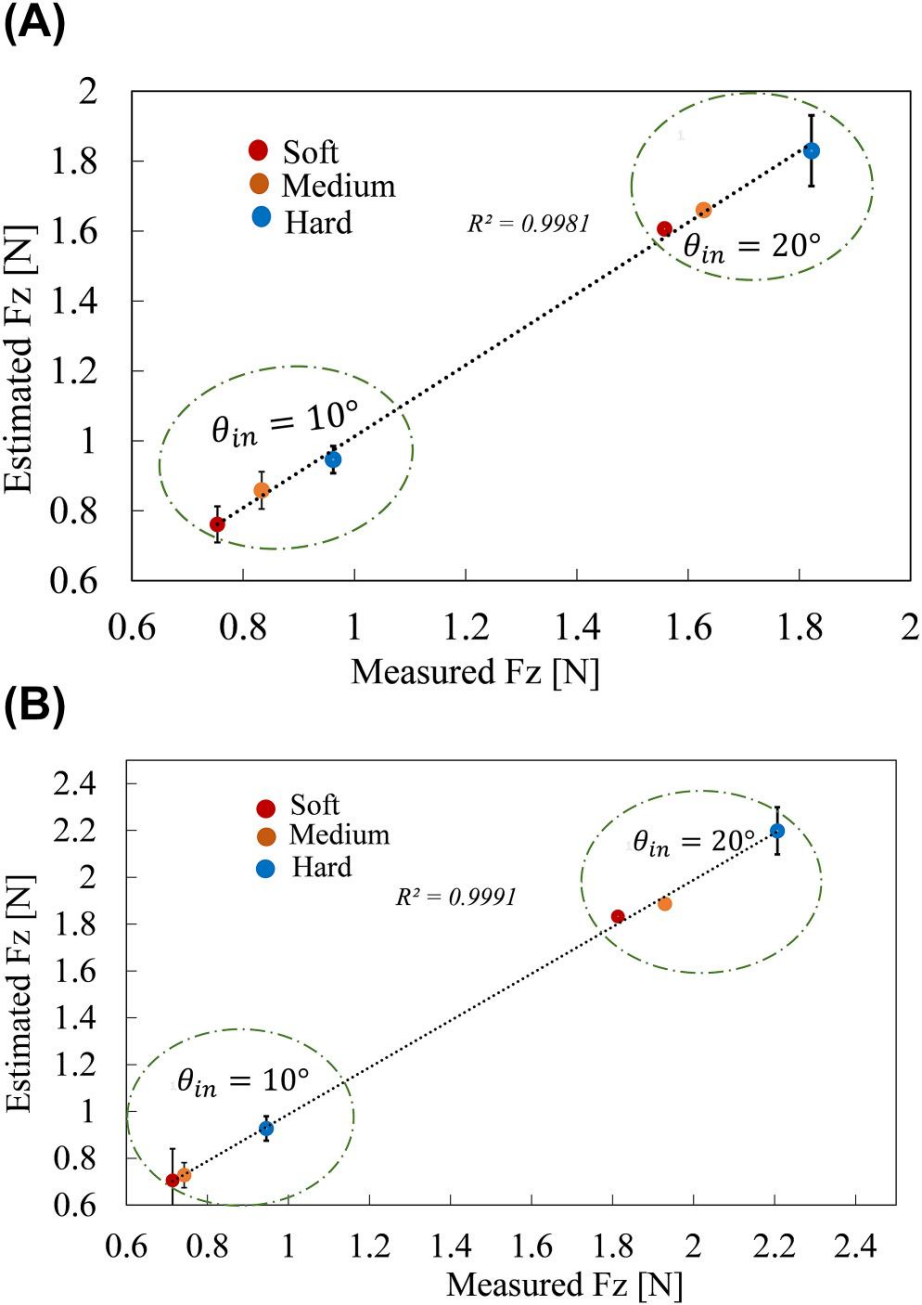
A relationship between the estimated and the measured average contact force on the three silicone phantoms under two palpation conditions; (A) ($\psi =0{}^{\circ},\theta_{c}=10{}^{\circ}$) and ($\theta_{in}=10{}^{\circ},20{}^{\circ}$); (B) ($\psi =0{}^{\circ},\theta_{c}=20{}^{\circ}$) and ($\theta_{in}=10{}^{\circ},20{}^{\circ}$). Three trials were conducted on each phantom and for each trial, the average contact force was calculated. The dotted straight lines show the linear fit of the estimated contact force to the measured contact force

The plots in Figure 12A,B show the relationship between the mean estimated contact force and the z‐component of the measured contact force for each sample under the considered palpation conditions. The results show a good linear relationship with *r = *0.99 in both cases. The estimated contact force values were relatively in good agreement with the measured values as confirmed by the maximum absolute error which was below 0.1 N. There was also a clear distinction between the contact force associated with the hardest sample and for the other two samples. For each initial contact angle condition (*θ*_*c*_ = 10°, *θ*_c_ = 20°), the largest contact force was recorded on the sample with the largest EM, 1.83 and 2.20 N respectively. The same was true for the other two samples. The EM of each sample was correlated with its corresponding contact force. Therefore, based on the sensed contact force response, the three samples can be distinguished from each other. However, for (*θ*_*c*_ = 20°, *θ*_in_ = 10°) condition, the contact force on the soft and medium samples was relatively close.

**FIGURE 13:**
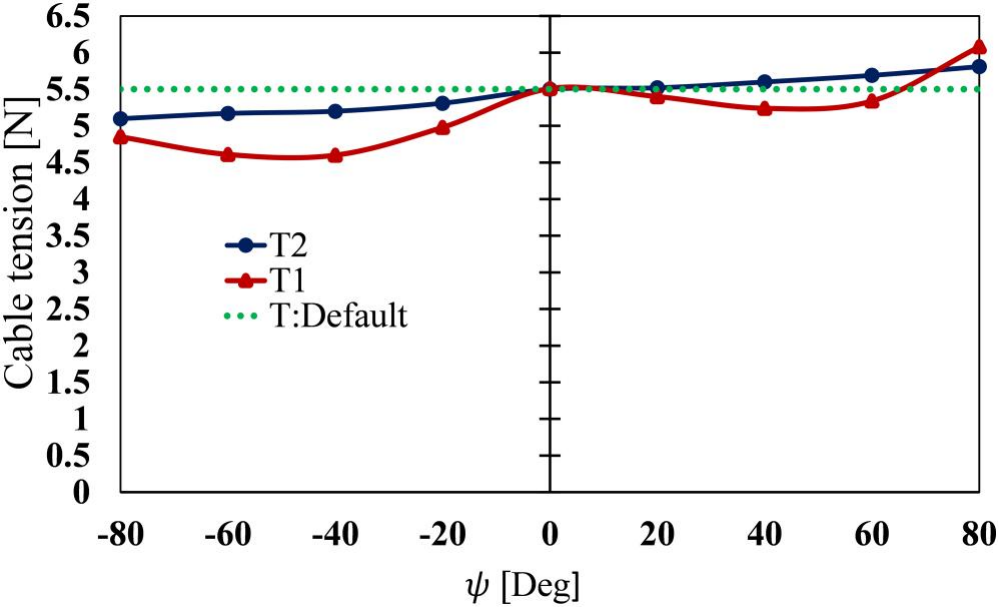
The effect of yaw motion on the pitch wire cable tension T1, and T2 tensioned at 5.5 N in home position. The pitch was at 0°. The *ψ* yaw was varied between ±80° in 20° increments

Figure 13 shows the effect of the yaw motion [−80°,80°] on the tension of the pitch cables, initially tensioned at 5.5 N in home position. Ideally, since the joints are mechanically decoupled the tensions T1 and T2 cables must remain constant at any arbitrary yaw angle, as indicated by the green dotted line. The results show relatively small changes in the cable tensions T1 and T2. The difference however, increased at [−80°,−40°] and [40°,80°] *ψ* regions. As *ψ* increased, the contact between the pitch cables and the 3D printed circular guide arcs also increased, resulting into the increased friction force between them. This may factor have contributed to the observed change in the cable tension. The maximum absolute error was 0.9 and 0.4 N for T1 and T2, respectively. It was deduced that the yaw motion had little effect on the pitch cable tension at any arbitrary *ψ* = ±80° and ultimately on the contact force estimation in the *z*‐direction. Contact force estimation experiments were conducted only at *ψ* = 0° and 20° conditions. At large yaw angles the using a *z*‐axis stage limited alignment of the tip with the ground truth loadcell under which silicone samples were mounted.

In this study, for each palpation condition on the soft and medium samples, there was an increase in the error when the indentation angle increased. An increase in the indentation angle resulted into an increase in the wire cable tension, and friction force in the joints and pulleys. This phenomena possibly may have contributed to the increase in the error. In this study, energy loss due to friction force was not considered. In an effort to improve this indirect contact force estimation method, future works should put into consideration the effect of friction on the contact force estimation accuracy as well as adopting a dynamic models.

## 4 DISCUSSION AND FUTURE WORK

The use of robotic systems for medical applications, such as in tumour localization and robotic‐assisted surgery is nontrivial. Robotic systems have the capability of providing objective and quantitative tactile information with high precision and reducing burden on physicians. This may greatly complement the manual palpation which is subjective in nature. Indirect contact force sensing methods based on robot dynamics and kinematics, cable tension observation, motor current, and machine learning have been a subject of interest with potential application in robot assisted surgery. Many studies have already demonstrated this feasibility in surgical robots with encouraging results.^22^, ^23^, ^24^ In this preliminary study, a static model was explored as a first step towards indirect contact force sensing for the proposed system, due to modelling simplicity, low joint velocity, and quasi static palpation conditions.

In this study, we proposed novel a 2DoF robotic palpation system for prostate palpation. Kinematic models for determining the position of the tip and joint angle estimation based on displacement measurement of the return springs were presented. The tip positioning and joint angle estimation results are promising. An indirect method to estimate the contact force between the tip of the proposed palpation probe and palpated sample based on the principle of virtual work was implemented. Finally, palpation experiments were conducted on silicone samples to confirm the feasibility of this contact force estimation method. There was consistence in agreement between the estimated and the ground truth contact force. The contact force was also related to the stiffness of the samples. It is therefore, suggested that this system holds potential for objective palpation of the prostate.

It should be noted that the proposed joint drive mechanism is not symmetrical in the forward and reverse pressing directions. Pressing against a sample in the forward direction by pulling the driving cable produces a relatively larger force than in the reverse direction when the return spring pulls the returning cable. The choice of the spring also has an influence on the largest possible pressing force in the reverse direction on the return spring side of the mechanism. Therefore, due to this non symmetric nature of the palpation system, to generate a large pressing force ideal for a deeper pressing depth, it is recommended that palpation is performed in the forward direction of the driving wire cable. The results in this study were based on the forward pressing direction. The returning spring side of the drive mechanism was used for estimating the joint angle by measuring the change in the length of the return spring using a distance sensor.

For the vertical position setup of the sample in the contact force estimation experiments, the predominant contact force was in the *z*‐direction, which in this study was estimated from the pitch joint torque and the Jacobian. Hence the study only investigated this particular contact force component. However, for other sample position setups, such as positioning the sample on either the left or right hand side of the tip, the yaw joint torque would be essential for providing a pressing force. In that case the need for force sensing on the yaw axis would be essential.

The focus of this study was on contact force and tip position estimation. In indentation tests, contact force, indentation information, size and the geometry of the indenter are essential parameters for estimating the stiffness of a sample. An extension of this study to include indentation depth measurement in order to estimate the stiffness, for example, using Hertz contact model will be undertaken. There is the need for miniaturisation of both the sensor unit and driving mechanism unit in order to make the system more compact enough to be handheld or mounted on a robotic manipulator for more dexterity. There is also the need to improve of the contact force estimation method by considering the robotic dynamics and the effect of friction in the system especially at larger angles. Lastly, a method to obtain a spatial distribution of the contact force as well as a stiffness map display will be developed.

## CONFLICT OF INTEREST

The authors have no conflicts of interest in relation to this article.

## Data Availability

Data available on request from the authors.
